# Cisplatin and Irinotecan as First-Line Chemotherapy for Previously Untreated Metastatic Thymic Carcinoma: Updated Analysis

**DOI:** 10.3389/fonc.2021.779700

**Published:** 2022-01-14

**Authors:** Akito Fukuda, Yusuke Okuma, Taiki Hakozaki, Kie Mirokuji, Makiko Yomota, Tsunekazu Hishima, Yukio Hosomi

**Affiliations:** ^1^ Department of Thoracic Oncology and Respiratory Medicine, Tokyo Metropolitan Cancer and Infectious Diseases Center Komagome Hospital, Tokyo, Japan; ^2^ Department of Thoracic Oncology, National Cancer Center Hospital, Tokyo, Japan; ^3^ Department of Pathology, Tokyo Metropolitan Cancer and Infectious diseases Center Komagome Hospital, Tokyo, Japan

**Keywords:** thymic carcinoma, cisplatin, irinotecan, first-line chemotherapy, metastasis

## Abstract

Platinum-based chemotherapy is the *de facto* standard treatment for metastatic or unresectable thymic carcinoma. The optimal chemotherapy regimen has not yet been determined, including whether this should be combined with a second- or third-generation anti-cancer agent. We retrospectively evaluated the data of patients with metastatic or unresectable thymic carcinoma who were treated with a combination of cisplatin and irinotecan as first-line chemotherapy between 2002 and 2021 (trial registration UMIN000012175). The primary endpoint was response rate according to the RECIST criteria version 1.1. Secondary endpoints were disease control rate, progression-free survival (PFS), overall survival (OS), and toxicity (adverse events). Some patients analyzed in this study were also included in the previous trial, which was terminated early. For this analysis, we included 18 patients with a median age of 56 years and an Eastern Cooperative Oncology Group performance status of 0 or 1. All patients had clinical stage IVa or IVb thymic carcinoma according to the Masaoka-Koga staging system. The response rate was 44% and the disease control rate was 89%. The median PFS was 8.4 months (95% confidence interval (CI): 2.7–11.6 months) and the median OS was 45.6 months (95% CI: 15.7–69.1 months). Grade 3 or worse hematological toxicity was observed in 5 patients and grade 3 or worse non-hematological toxicity was observed in 3 patients. None of the patients developed febrile neutropenia, and no treatment-related deaths occurred. Thus, the combination of cisplatin and irinotecan as first-line chemotherapy for metastatic thymic carcinoma showed efficacy and acceptable toxicity.

## Introduction

Thymic carcinoma is a rare cancer arising from the mediastinum originating from thymic epithelial cells, accounting for approximately 5% of all thymic epithelial tumors (1). Thymic carcinoma tends to metastasize with invasive growth, but without associated immunological symptoms such as type B thymoma; therefore, it is often diagnosed at an advanced stage and has a poor prognosis (2).

The standard of care for metastatic thymic carcinoma is palliative chemotherapy. The key drugs for treating thymic malignancies include platinum and doxorubicin containing chemotherapy used in the Einhorn protocol (3) including CAP (cisplatin, doxorubicin, cyclophosphamide, and prednisone) (4), CODE (cisplatin, vincristine, doxorubicin, and etoposide) (5), ADOC (cisplatin, doxorubicin, vincristine, and cyclophosphamide) (6), and VIP (etoposide, ifosfamide, and cisplatin) regimens (7, 8). Doxorubicin does not tend to be beneficial in the treatment of thymic carcinoma; therefore, a combination of carboplatin and paclitaxel is now commonly used owing to its satisfactory response rate, time-to-event data, and toxicity profile (9–11). However, given the rarity of this cancer, data are only available from phase II or retrospective studies with small sample sizes. There is minimal evidence of the effectiveness of first- or later-line chemotherapy for thymic malignancies. With respect to later lines of chemotherapy, recent phase II trials have shown an increase in the clinical effectiveness of cytotoxic chemotherapy, molecular-targeted agents, and immune checkpoint inhibitors. The key drugs or optimal strategy for the treatment of thymic carcinoma are gradually being revealed, but there is still ample room for development.

Our cancer center previously reported a retrospective single-center analysis that demonstrated the efficacy and mild hematological toxicity of combination treatment with cisplatin and irinotecan as the first-line chemotherapy for patients with metastatic/unresectable thymic carcinoma (12). We performed a phase II clinical trial of irinotecan and cisplatin for thymic carcinoma. Although the clinical trial was interrupted because of late accrual (UMIN000012175), we reported all treated patients with irinotecan and cisplatin for thymic carcinoma. The purpose of the current study was to evaluate the efficacy and toxicity of this combination therapy as first-line chemotherapy for irinotecan and cisplatin for metastatic/unresectable thymic carcinoma by including additional cases over a longer follow-up time.

## Patients and Methods

### Study Cohort and Data Acquisition

This retrospective observational study included patients with metastatic/unresectable thymic carcinoma who received a combination of cisplatin and irinotecan as first-line chemotherapy between January 2002 and December 2021 at Tokyo Metropolitan Cancer and Infectious Disease Center Komagome Hospital. All patients had histologically confirmed stage IVa or IVb thymic carcinoma based on the Masaoka-Koga staging system (13). The inclusion criteria were as follows: <75 years of age, Eastern Cooperative Oncology Group Performance Scale status (ECOG PS) of 0 or 1, adequate organ function for chemotherapy, neutrophil count of 1500 cells/mm³ or higher, hemoglobin concentration of 9.0 g/dL or higher, platelet count of 10.0 × 10⁴/mm^3^ or more, and adequate renal and liver function. Patients with resectable tumors and those who had previously undergone treatment were excluded. The Institutional Review Board of Komagome Hospital approved the present study (IRB number 2415), and the study protocol adhered to the principles of the Declaration of Helsinki.

Between October 30, 2013 and March 22, 2019, our cancer center performed a prospective phase II trial of cisplatin and irinotecan for patients with previously untreated thymic carcinoma; however, the trial had to be terminated because of late accrual. For the current analysis, further patients were enrolled in the trial (UMIN000012175) who were treated with cisplatin and irinotecan, thereby enabling data collection according to the same study protocol. This prospective study has also been approved by the Institutional Review Board of Komagome Hospital (IRB number 1306). 

### Treatment Procedure

The included patients were treated with combination chemotherapy of cisplatin (60 mg/m^2^ or 80 mg/m^2^) on day 1 and irinotecan (60 mg/m^2^) on days 1, 8, and 15 every 4 weeks for up to 6 cycles if the patients did not exhibit unacceptable toxicities or disease progression. The dose of cisplatin was reduced from 80 mg/m^2^ to 60 mg/m^2^ at the physician’s discretion.

### Evaluation and Statistical Analysis

The primary endpoint of this study was response rate based on the Response Evaluation Criteria in Solid Tumors (RECIST) version 1.1 (14). Objective response was defined as a complete response plus partial response, and disease control was defined as an objective response plus stable disease. The secondary endpoints were progression-free survival (PFS), overall survival (OS), and disease control rate (DCR) to assess the efficacy and toxicity of this regimen. PFS was assessed from the date of the first chemotherapy cycle until the date of progressive disease first detected by the investigators’ assessments or loss to follow-up. OS was assessed from the date of the first cycle of chemotherapy until death or loss to follow-up. Disease assessment was evaluated by computed tomography after every two cycles of chemotherapy. After the last chemotherapy cycle, computed tomography was performed once every two to three months until the disease progressed.

Safety was assessed according to the Common Terminology Criteria for Adverse Events version 4.0 (CTCAE v4.0). We also collected and evaluated data on second- and later-line chemotherapy.

All statistical analyses were performed using the JMP 14.3.0 statistical software for Windows (SAS Institute, Cary, NC) with a two-sided α value set at 0.05.

## Results

### Patient Characteristics

Eighteen patients with previously untreated metastatic/unresectable thymic carcinoma were treated with irinotecan and cisplatin. One of eighteen patient has been enrolled in the prospective analysis (trial registration UMIN000012175). Clinical characteristics of the patients are summarized in **Table 1**. There were 7 patients (39%) with an ECOG PS of 0 and 11 (61%) with an ECOG PS of 1. Three patients (17%) had Masaoka-Koga stage IVa and fifteen (83%) had stage IVb disease. The most common metastatic site was the lungs. With respect to the pathological diagnosis, 14 patients (77%) had squamous cell carcinoma, 3 (17%) had undifferentiated carcinoma, and 1 (6%) had large cell neuroendocrine carcinoma. There were no instances of paraneoplastic syndrome or immunological complications, including myasthenia gravis, pure red cell anemia, or Good syndrome.

**Table 1:** Patient characteristics.

Patient characteristics	Value (N = 18)
Age (years), median (range)	56 (44–73)
Sex, n (%)	
Male	11 (61)
Female	7 (39)
ECOG	
0	7 (39)
1	11 (61)
2-4	0 (0)
Masaoka-Koga staging system	
IVa	3 (17)
IVb	15 (83)
Metastatic sites at diagnosis	
LUNG	7
HEP	4
LYM	6
BRA	2
OSS	5
PLE	8
Histology	
Squamous cell carcinoma	14 (77)
Undifferentiated carcinoma	3 (17)
Large cell neuroendocrine carcinoma	1 (6)
Smoking history	
Never-smoker	5 (28)
Previously/current smoker	13 (72)
Paraneoplastic syndrome	0

Data are presented as n (%) unless otherwise stated. ECOG, Eastern Cooperative Oncology Group; HEP, liver; LYM, lymph nodes; BRA, brain; OSS, bone; PLE, pleura.

### Treatment Delivery

**Figure 1:**
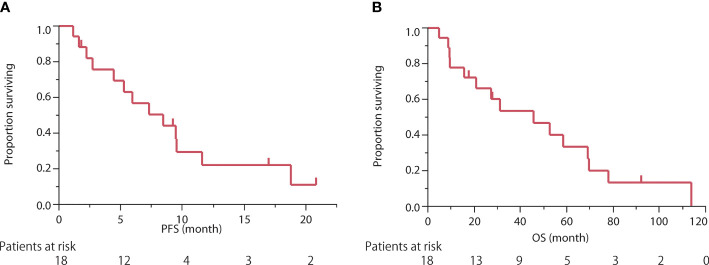
**(A)** Swimmer’s plot of progression-free survival in individual patients at each chemotherapeutic line. **(B)** Change in tumor size from baseline (%) in thymic carcinoma patients treated with the cisplatin and irinotecan combination regimen as the first-line chemotherapy. TC, carboplatin and paclitaxel; GV, gemcitabine and vinorelbine; DOC, docetaxel; AMR, amrubicin; CPT-11, irinotecan; PEM, pemetrexed; ADOC, cisplatin, doxorubicin, vincristine, and cyclophosphamide; GEM, gemcitabine; CBG, carboplatin and gemcitabine; CA, cisplatin and adriamycin; PE, cisplatin and etoposide.

The delivery mode for the irinotecan and cisplatin treatment is summarized in **Table 2**. Cisplatin was administered at a dose of 80 mg/m^2^ in 3 patients (17%) and 60 mg/m^2^ in 15 (83%). The average number of cycles of cisplatin and irinotecan combination therapy was 3.6 (range, 1–6). Two patients discontinued chemotherapy due to progressive disease, and three patients discontinued chemotherapy because of adverse events. The average number of later lines of chemotherapy was 2.8. S-1, carboplatin, and paclitaxel combination therapies were commonly used in the second-line setting (**Figure 1A**). One patient received nivolumab as part of an investigation with the PREMIER (15) study and one patient received lenvatinib through the REMORA study (16).

**Table 2:** Delivery methods of first- and later-line chemotherapy and response to cisplatin and irinotecan combination therapy.

Patient characteristics	Value (N = 18)
Cisplatin dose	
80 mg/m^2^	3 (17)
60 mg/m^2^	15 (83)
Average number of cycles	3.6 (1–6)
Response to cisplatin and irinotecan	
Complete response	0 (0)
Partial response	8 (44)
Stable disease	8 (44)
Progressive disease	2 (12)
Discontinuation of first-line regimen	6 (33)
Reason for discontinuation	
Progressive disease	2
Adverse event	3
Other	1
Later chemotherapy line	
Average of later chemotherapy line (range)	2.8 (0–8)
Number of later chemotherapy line	1
8	1
6	1
5	3
4	3
3	3
2	4
1	2
0	

Data are presented as n (%) unless otherwise stated.

### Treatment Efficacy

**Figure 2:**
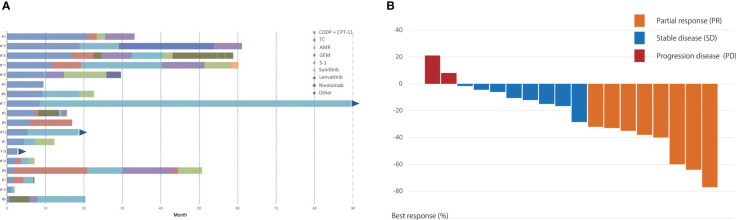
**(A)** Progression-free survival and **(B)** overall survival for 18 patients with unresectable thymic carcinoma who received cisplatin and irinotecan combination therapy.

Among the 18 patients, 8 (44%) had a partial response and 8 (44%) had stable disease. The objective response and disease control rates were 44% and 89%, respectively. Progressive disease was observed in only two patients (**Table 2**). The best response and change in tumor size for each patient are shown in **Figure 1B**. Only 2 of the 18 patients exhibited tumor growth based on baseline measurements. Eight patients showed a tumor reduction rate of 30% or more. The median follow-up time was 29.5 months. The median PFS was 8.4 months [95% confidence interval (CI): 2.7–11.6 months] (**Figure 2A**). The median OS was 45.6 months (95% CI: 15.7–69.1 months) (**Figure 2B**).

A Swimmer plot is shown in **Figure 1A**. Fifteen patients received S-1 as the second- or later-line regimen, with a response rate of 33% and median PFS of 8.1 months (95% CI: 0.7–10.3 months) (**Table 3**). Eight patients received carboplatin and paclitaxel as the second- or the later-line regimen, with a response rate of 38% and median PFS of 4.1 months (95% CI: 0.9–7.7 months). One patient (number 17) was treated with nivolumab as third-line chemotherapy, with a PFS of 25 months. One patient (number 14) received lenvatinib as the fifth-line chemotherapy with a PFS of 3.4 months.

**Table 3:** Efficacy of second- or later-line regimen.

Chemotherapy	N	PR	SD	PD	RR (%)	Median PFS, months (95% confidence interval)
S-1	15	5	5	5	33%	8.1 (0.7–10.3)
CBDCA+ PTX	8*	3	1	3	38%	4.1 (0.9–7.7)

PFS, progression-free survival; PR, partial response; SD, stable disease; PD, progressive disease; RR, response rate; CBDCA, carboplatin; PTX, paclitaxel.

*The outcome of one patient was not evaluated.

### Toxicities

The toxicity results are shown in **Table 4**. Grade 3 hematological toxicity was observed in six patients. The major treatment-related adverse events were nausea (73%), neutropenia (72%), and leukocytopenia (45%). Three patients showed grade 3 neutropenia; however, none of the patients developed febrile neutropenia. Grade 3 non-hematological adverse events were observed in five patients and grade 3 diarrhea was observed in one patient. No patient exhibited Grade 4 adverse events and there were no treatment-related deaths. Three patients discontinued the cisplatin and irinotecan regimens because of gastrointestinal adverse events.

**Table 4:** Treatment-related hematological and non-hematological adverse events.

Adverse events	Grade 1 or 2, n (%)	Grade 3, n (%)	Grade 4, n (%)
Hematological			
Leukocytopenia	7 (39)	1 (6)	0
Neutropenia	9 (50)	3 (17)	0
Anemia	6 (33)	2 (11)	0
Thrombocytopenia	3 (16)	0	0
Febrile neutropenia	–	0	0
Non-hematological			
Nausea	12 (67)	1 (6)	0
Vomiting	5 (27)	0 (0)	0
Anorexia	4 (22)	3 (17)	0
Diarrhea	5 (29)	1 (6)	0

## Discussion

In the current analysis, our long-term experience with cisplatin and irinotecan combination chemotherapy for unresectable or metastatic thymic carcinoma demonstrated clinical effectiveness and tolerability, which is in line with previous studies (**Table 5**). It is worth noting that the 5-year survival rate for patients with metastatic or recurrent thymic carcinoma in this trial was 26.8%, and the 1-year PFS rate was 22.1%.

**Table 5:** Previously reported platinum-combination chemotherapy for unresectable/metastatic thymic carcinoma.

Study	Chemotherapy	N	RR (%)	PFS, months (median)	OS, months (median)	Toxicities (Grade ≥ 3)	
						Hematological (%)	FN (%)
Agatsuma et al. (6)	ADOC	34	50	−	21.3	71	12
Magois et al. (8)	VIP	9	44	−	20	−	−
Igawa et al. (9)	CBDCA+PTX	11	36	7.9	22.7	45	0
Hirai et al. (10)	CBDCA+PTX	39	36	7.5	−	44	5
Current study	CDDP+CPT-11	18	44	8.4	45.6	33	0

PFS, progression-free survival; RR, response rate; OS, overall survival; FN, febrile neutropenia; ADOC, cisplatin + doxorubicin + vincristine + cyclophosphamide; VIP, cisplatin + etoposide + cyclophosphamide; CBDCA, carboplatin; PTX, paclitaxel; CDDP, cisplatin; CPT-11, irinotecan.

The National Comprehensive Cancer Network (NCCN) Clinical Practice Guidelines in Oncology (17) lists platinum-containing agents (including carboplatin, paclitaxel, ADOC, VIP, and CAP) as the recommended first-line chemotherapy options for thymic carcinoma. The guideline also suggests carboplatin and paclitaxel combination therapy as a *de facto* standard first-line chemotherapy, owing to its mild toxicity but similar efficacy. The present study also showed that cisplatin and irinotecan combination therapy had good efficacy and mild toxicity; moreover, the rate of grade 3 or 4 toxicities was 33% in the present study. However, it was more than 40% in the other regimens (**Table 5**). This combination appears to have lower toxicity than the carboplatin and paclitaxel combination regimen given the lack of peripheral neuropathy and that the patients can survive for more than 5 years. Thus, fewer adverse events could be a reason for choosing a cisplatin-based regimen. In terms of efficacy, cisplatin irinotecan therapy, as the first-line regimen, showed positive activity for thymic carcinoma. The ORR and PFS were 44% and 8.4 months, respectively. Xue et al. reported the efficacy of gemcitabine and platinum regimen as a first-line chemotherapy for stage IV thymic carcinoma (11). The PFS was 12.0 months, which seems long; however, 64.5% of patients treated with gemcitabine and platinum regimens received radiotherapy after chemotherapy. Furthermore, the ORR of the gemcitabine and platinum regimens was 29%. Therefore, it is not possible to conclude that the gemcitabine and platinum regimens are better than cisplatin and irinotecan regimens. Additionally, we also believe that irinotecan is a key drug for treating thymic malignancies. Antibody-drug conjugates with topoisomerase I inhibitor (irinotecan) payload are potentially going to be active agents for thymic malignancies. Moreover, cisplatin and irinotecan combination therapy showed therapeutic activity as a second-line regimen, with a response rate of 29% and mild toxicity (18). These results suggest that irinotecan and cisplatin combination therapy may be a useful regimen for advanced thymic carcinoma.

Based on the results of a randomized controlled phase 3 trial performed in Japanese patients (JCOG9511) (19), cisplatin and irinotecan combination therapy had been widely used as a first-line regimen for extensive-disease small-cell lung cancer (ED-SCLC) in Japan until the era of oncoimmunotherapy. In the JCOG9511 trial, the cisplatin and irinotecan regimen resulted in longer survival and milder hematological toxicity than the cisplatin and etoposide regimen for ED-SCLC. After the results of the JCOG9511 trial were published, a randomized controlled trial (SWOG S0124) in North American patients (20) failed to show the superiority of cisplatin and irinotecan chemotherapy for ED-SCLC over cisplatin and etoposide, as both exhibited comparable efficacy. The toxicity profiles, including diarrhea, differed in the cisplatin and irinotecan regimen arms in the SWOG S0124 and JCOG9511 trials, but there were no significant differences in treatment delivery between the two arms. Therefore, cisplatin and irinotecan combination therapy is not suggested as a suitable treatment option for North American patients with small-cell lung cancer, whereas it is feasible in Japanese patients in terms of tolerability.

In the present study, the response rate, PFS, and OS of cisplatin and irinotecan chemotherapy for thymic carcinoma were 44%, 8.4 months, and 45.6 months, respectively. In the previous study on carboplatin and paclitaxel, the response rate, PFS, and OS were 36%, 7.9 months, and 22.7 months, respectively (9). We did not conduct a comparative study; therefore, this was only compared with published data, the PFS in the present study was similar to that in the study on carboplatin and paclitaxel regimen. In contrast, the response was higher, and the median OS was longer than as compared to the that in the study on carboplatin and paclitaxel. With regard to platinum-based treatments, it is controversial whether cisplatin- or carboplatin-based chemotherapy treatment is preferred for patients with thymic malignances (21, 22). These studies showed that a cisplatin-based regimen was superior to a carboplatin-based regimen with respect to the response rate and OS. However, carboplatin tended to be used in elderly patients and in patients with low performance status, and there has been no comparative trial or clear survival data. We speculate that the reason for the longer OS observed in this study was the inclusion of post-treatment regimens and the ability of patients to register in other clinical trials, such as carboplatin and paclitaxel combination therapy, S-1 (23), lenvatinib (16), and nivolumab (15). In particular, 15 patients who received S-1 showed a longer PFS (8.1 months), and 3 of these 15 patients had a long-term response of over 10 months. A recent phase II trial of S-1 as a second- or later line-regimen for 23 patients with thymic carcinoma demonstrated moderate activity, resulting in a 30.8% response rate (90% CI, 18.3–46.9), an 80.8% disease control rate (90% CI, 65.4–90.3), a median PFS of 4.3 months (95% CI, 2.3–10.3 months), and a median OS of 27.4 months (95% CI, 16.6–34.3) (23). The present study also supports the effectiveness of S-1 as a second- or later-line chemotherapy.

In the current era of molecular-targeted drugs and immuno-oncology, focusing only on a cytotoxic chemotherapy regimen is no longer necessary. Lenvatinib, a multi-kinase inhibitor that inhibits receptor tyrosine kinases such as VEGFR1 (FLT1), VEGFR2 (KDR), VEGFR (FLT4), FGFR, PDGFR, KIT, and RET FGR1, showed an objective response of 38% and a disease control rate of 95% (16). Sunitinib, a multitargeted kinase inhibitor that inhibits certain receptor tyrosine kinases, has been reported to have a moderate effect on thymic carcinoma (24). Pembrolizumab demonstrated a mild objective response (23%) with several long responders (25). Currently, chemoimmunotherapy is considered to be the optimal strategy for non-small cell lung cancer (26–30) and small-cell lung cancer (31, 32). The biological plausibility of targeted drugs for thymic carcinoma is currently unknown because there is no known biomarker for thymic carcinoma. It is important to identify the oncogenic drivers for lenvatinib and a biomarker for selecting patients that would benefit from pembrolizumab and to reduce the immunological toxicities of immunotherapy. The present milestone of chemotherapy is based on single agents for second-line chemotherapy and platinum-containing cytotoxic chemotherapy for first-line chemotherapy. In the future, finding the best combination regimen with key drugs in first-line therapy is crucial, even in rare cancers. In fact, the paradigm of immunochemotherapy focuses on the combination of other agents, including lenvatinib, in non-squamous non-small cell lung cancer (MK-7902-006/E7080-G000-315/LEAP-006) [NCT04716933]. Irinotecan and cisplatin could be considered as key drugs in combination therapy with immunotherapy or molecular-targeted drugs based on the present and previous studies.

The present study had several limitations. First, this study was a retrospective analysis of phase 2 trial data obtained from a single center. Second, we evaluated only a small number of patients. The broad range of 95% confidence interval was due to the small sample size. However, this is an unavoidable limitation as thymic carcinoma is a rare disease; therefore, the sample sizes for such studies are generally small. Moreover, given the current direction of the field with an increasing focus on immuno-chemotherapy, a detailed discussion of conventional chemotherapy for thymic malignancies is no longer be relevant. Thymic carcinoma is characterized by high expression of PD-L1; thus, immune checkpoint inhibitors may also be a key drug for this malignancy. Combination therapies including lenvatinib, pembrolizumab, or sunitinib for patients previously treated with chemotherapy for thymic carcinoma are currently being investigated, and chemoimmunotherapy is expected to become the first-line treatment in the future. However, further biological investigations to identify the origins of thymic carcinoma and potential actionable targets must continue to conquer this disease.

In summary, the combined use of cisplatin and irinotecan as first-line chemotherapy for metastatic or recurrent thymic carcinoma revealed efficacy and acceptable toxicity. Therefore, we propose that this combination chemotherapy is a feasible option as first-line chemotherapy for thymic carcinoma. However, it is important to further investigate combination chemotherapies and immunotherapies for thymic malignancies.

## Data Availability Statement

The raw data supporting the conclusions of this article will be made available by the authors, without undue reservation.

## Ethics Statement

The Institutional Review Board of Komagome Hospital approved the present study (IRB number 2415). Written informed consent from the participants’ legal guardian/next of kin was not required to participate in this study in accordance with the national legislation and the institutional requirements.

## Author Contributions

AF: Writing Original Draft, Data Curation, Formal analysis. YO: Conceptualization, Methodology, Data Curation and Writing and Editing Draft. THa: Writing- Reviewing and Editing, Data Curation, Investigation. KM Writing- Reviewing and Editing, Data Curation, Invetsitgation. MY: Writing- Reviewing and Editing, Investigation. THi: Writing- Reviewing and Editing, Investigation. YH: Writing- Reviewing and Editing, Investigation. All authors contributed to the article and approved the submitted version.

## Conflict of Interest

The authors declare that the research was conducted in the absence of any commercial or financial relationships that could be construed as a potential conflict of interest.

## Publisher’s Note

All claims expressed in this article are solely those of the authors and do not necessarily represent those of their affiliated organizations, or those of the publisher, the editors and the reviewers. Any product that may be evaluated in this article, or claim that may be made by its manufacturer, is not guaranteed or endorsed by the publisher.
